# Inhomogeneous Fluid Motion Induced by Standing Surface Acoustic Wave (SAW): A Finite Element Study

**DOI:** 10.3390/mi17030330

**Published:** 2026-03-06

**Authors:** Jialong Hu, Chao Zhang, Yufeng Zhou

**Affiliations:** 1 State Key Laboratory of Ultrasound in Medicine and Engineering, College of Biomedical Engineering, Chongqing Medical University, Chongqing 400016, China; hjl35105988@163.com (J.H.); zhangch11860@163.com (C.Z.); 2 Chongqing Key Laboratory of Biomedical Engineering, Chongqing Medical University, Chongqing 400016, China; 3 National Medical Products Administration (NMPA) Key Laboratory for Quality Evaluation of Ultrasonic Surgical Equipment, 507 Gaoxin Ave., Donghu New Technology Development Zone, Wuhan 430075, China

**Keywords:** surface acoustic wave (SAW), inhomogeneous fluid, relocation, fluid mixing, acoustic radiation force (ARF), streaming velocity

## Abstract

Acoustofluidics has emerged as a transformative technology for contact-free manipulation of microparticles and fluids in microscale systems. Although bulk acoustic waves (BAWs) are known to displace inhomogeneous fluids through acoustic radiation force acting at fluid interfaces, the capability of surface acoustic waves (SAWs) to produce analogous relocation phenomena remains largely unexplored. This study addresses a critical gap in acoustofluidic theory by presenting the first comprehensive finite element method investigation of SAW-driven motion of inhomogeneous fluid confined within microchannels of widths equal to one full or one-half SAW wavelength. Unlike BAW-based system that generate uniform pressure fields across channel heights, SAW devices exhibit inherently nonuniform vertical pressure distributions and intense near-boundary streaming—features that fundamentally alter fluid relocation dynamics. Our simulations demonstrate that despite high-frequency operation (6.65 MHz) and strong ARF, standing SAW fields fail to achieve stable fluid relocation in both initially stable and unstable configurations due to vertical pressure stratification and rapid floor-level streaming. Nevertheless, these same characteristics generate vigorous transverse folding flows that enable exceptionally rapid homogenization, offering a distinct acoustofluidic mechanism for on-chip mixing. These findings not only elucidate fundamental physical differences between BAW and SAW actuation in multiphase microfluidic systems but also establish design principles for SAW-induced microfluidic mixers. The results provide crucial theoretical guidance for device optimization where rapid homogenization is desired over stable stratification.

## 1. Introduction

Emerging acoustofluidic technologies integrate acoustics with microfluidic platforms to manipulate particles and fluids in a contactless, label-free, and biocompatible manner. Over the past decade, this synergy has experienced significant growth across diverse biological and biomedical applications, ranging from the deterministic patterning of heterogeneous cells for tissue engineering to the on-chip automated isolation of biological substances (e.g., cells, viruses, biomolecules, exosomes, and extracellular vesicles) from biofluids for rapid, point-of-care diagnostics [1,2,3]. Acoustic waves propagate through the medium carrying the targets while simultaneously exerting forces on both the medium and any objects encountered along their path. Two primary wave modalities are utilized in practical devices: bulk acoustic wave (BAW) and surface acoustic wave (SAW). A typical BAW device comprises a piezoelectric transducer and a resonant chamber, whereas SAW requires patterned electrodes on top of a piezoelectric substrate. Both modalities have demonstrated extraordinary versatility and effectiveness.

Beyond particle manipulation, the acoustic radiation force (ARF) acts perpendicular to the interface between two coflowing fluids, whether miscible or immiscible, whenever their acoustic impedances differ by as little as 0.1% [4]. The interface migrates until the fluid with higher acoustic impedance resides at the pressure node, yielding a final stable stream configuration. BAW devices can exploit this response to extract blood plasma, yet extracting cells from whole blood into a central saline buffer solution remains challenging because even small discrepancies in acoustic impedance can trigger bulk relocation [5]. For inhomogeneous laminated flows in an initially unstable configuration with a low-impedance stream positioned at the center or nodal plane, the higher-impedance streams gradually broaden, eventually enclosing the entire microchannel and stabilizing the resulting density profiles at the pressure node where acoustic pressure (1–100 Pa) outweighs hydrostatic pressure differences (~1 Pa) [4,6,7]. This phenomenon in the acoustofluidic microchannel is termed relocation. By judiciously tuning the acoustic contrast between the two liquids, the performance of acoustophoretic washing of beads or cells can be substantially improved [4]. Conversely, positioning the high-impedance stream at the node suppresses interface broadening by damping streaming. This insight underpins iso-acoustic focusing (IAF), which separates cell types according to their acousto-mechanical properties and overcomes the essential size dependency limitation of acoustophoresis—representing an acoustic analog to density gradient centrifugation or iso-electric focusing [8]. To maneuver objects below the classical 2 µm size limit for acoustic focusing, gradient acoustic focusing (GAF) imposes a stabilizing volume force that counteracts acoustic streaming [9], enabling sub-micrometer particles to transverse from one medium to another in continuous flow. This strategy has already achieved efficient medium exchange of clinically relevant bacterial species (e.g., *Staphylococcus aureus*, *Streptococcus pneumoniae*, and *Escherichia coli*) and their separation from lysed blood on a chip-integrated system [9].

SAWs (Rayleigh, Love, or Lamb mode) travel along the surface of an elastic substrate, decaying exponentially within 1–2 wavelengths [10]. SAW devices are particularly attractive for acoustofluidics because of their high biocompatibility, versatile functionalities, rapid fluid actuation, contactless particle manipulation, and facile integration with other microfluidic components and mass production through standard mechanical–electrical micro-system (MEMS) fabrication protocols [11]. Energy is concentrated with a shallow substrate layer, yielding superior solid–fluid coupling and higher energy conversion efficiency compared with typical BAW resonators [12]. These advantages have enabled applications in rapid fluid pumping, contactless particle manipulation, and ultrafast acoustic micromixing for chemical synthesis, nanoparticle fabrication, cell culture, biochemical analysis, and cell lysis [13]. For example, a vertical-type SAW acousto-microfluidic mixer achieves lysis of >90% blood cells at a high throughput of ~0.2 mL/min but at a low exciting voltage of 6.0 V_rms_ [14]. Recent advances in SAW-based microfluidics have expanded to complex fluid handling. For fluid manipulation, SAWs have demonstrated rapid micro-mixing through acoustic streaming [13,14], droplet generation and nebulization [15], and localized heating for chemical synthesis [16]. Notably, phase separation of nonionic surfactant solutions has been demonstrated using standing SAW (SSAW) [17], while the comparison of mixing efficiency between standing and traveling SAW modes revealed that double-vortex patterns in SSAW fields enhance folding-based mixing [18]. However, these studies primarily addressed homogeneous fluids or particle-laden suspensions; the behavior of inhomogeneous, stratified fluids under SAW actuation—particularly regarding interface stabilization and relocation—remains theoretically unresolved. This gap is significant because many biomedical applications (e.g., cell washing, medium exchange, density-based separation) rely on controlled manipulation of fluid interfaces with distinct acoustic impedances.

Our prior numerical investigation revealed that the microchannel aspect ratio (AR) critically determines BAW-induced stability and relocation of inhomogeneous miscible fluids [19]. In a stable configuration, the high-impedance fluid in the central region tends to sink under gravity as the microchannel height increases. In contrast, fluid flow in an unstable configuration exhibits greater complexity. The relocation process, particularly the mass fraction of the high-impedance fluid, varies with the number of Rayleigh streaming rolls in the microchannel with an increasing height. In this study, we extended this analysis to standing SAW fields. Using the finite element method (FEM) simulation, we explore the possibility of inhomogeneous fluid relocation induced by a standing SAW field for both initially stable and unstable configurations. Cross-sectional flow maps elucidate the roles of the vertically nonuniform acoustic pressure distribution and the intense Rayleigh streaming near the substrate in this phenomenon. Microchannels with widths equal to either one full or one-half wavelength of the SAW field were selected for simulation, and the resulting patterns of inhomogeneous fluid motion were compared with those induced by BAWs. Finally, we evaluated the performance of SAW-induced fluid mixing to assess its potential for future microfluidic applications.

## 2. Materials and Methods

**Figure 1:**
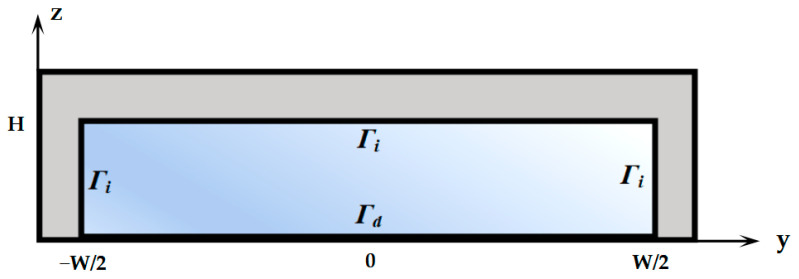
Simplified simulation domain of a standing SAW in a PDMS microfluidic channel.

To simplify the numerical calculation, impedance boundary conditions were imposed on the side walls and ceiling of the polydimethylsiloxane (PDMS) microchannel to account for acoustic energy dissipation, while a prescribed displacement function on the piezoelectric substrate supplied the standing SAW excitation [20]. The rectangular microfluidic channel with a width of *W* = 600 μm and a height of *H* = 125 μm, as shown in Figure 1, served as the calculation domain. Two side walls and the ceiling of the PDMS microchannel were denoted by the boundary condition of Γ*_i_*, while a displacement boundary condition of Γ*_d_* was applied to its substrate–fluid interface (floor).

(1) $$n\cdot \nabla p_{1}=i\frac{\omega \rho_{0}}{\rho_{m}c_{m}}p_{1},$$

(2) $$v_{1}\left(t,y\right)=\frac{\partial u\left(t,y\right)}{\partial t},$$

where *ρ*_0_ is the fluid density; *ρ_m_* and *c_m_* are the density and sound speed of the PDMS material, respectively; *n* is the outward normal vector; and *u* (*t*, *y*) describes the temporally and spatially dependent surface motion. The particle displacements induced by SAW perpendicular and parallel to the propagation direction are given by [20],

(3) $$\begin{array}{c} u_{y}\left(t,y\right)=0.6u_{0}e^{-C_{d}y}\left\{sin\left[\frac{-2\pi \left(y-W/2\right)}{\lambda }+\omega t-\Delta \phi \right]\right\}\\ +sin\left[\frac{-2\pi \left(W/2-y\right)}{\lambda }+\omega t\right]\\ u_{z}\left(t,y\right)=u_{0}e^{-C_{d}y}\left\{cos\left[\frac{-2\pi \left(y-W/2\right)}{\lambda }+\omega t-\Delta \phi \right]\right\}\\ +cos\left[\frac{-2\pi \left(W/2-y\right)}{\lambda }+\omega t\right] \end{array},$$

where *u*_0_ is the displacement amplitude; *C_d_* is the displacement decay coefficient; *W* is the channel width; *ω* is the angular frequency; and Δ*ϕ* is the initial phase.

Assuming constant acoustic energy density and negligible first-order field perturbations, the volumetric acoustic radiation force was calculated using the expression below for a significant reduction in computation.

(4) $$\begin{array}{c} f_{ac}=-\nabla \cdot <\rho_{0}v_{1}v_{1}>=f_{1}+f_{2}+f_{3}\\ f_{grad}=\frac{1}{2}\nabla (\kappa_{0}<\;{\left|p_{1}\right|}^{2}>-\rho_{0}<\;{\left|v_{1}\right|}^{2}>)\\ f_{str}=\;<v_{1}\times \nabla \times \left(\rho_{0}v_{1}\right)>\\ f_{rl}=-\frac{1}{2}<\;{\left|p_{1}\right|}^{2}>\nabla \kappa_{0}-\frac{1}{2}<\;{\left|v_{1}\right|}^{2}>\nabla \rho_{0} \end{array},$$

All material properties and acoustic parameters used in the simulation are listed in Table 1.

This study will specifically address inhomogeneous fluids without suspended particles; that is, as stratified layers of miscible fluids with distinct acoustic impedances (a Ficoll PM70 solution with a mass fraction of 0.1 served as the high-impedance fluid, while deionized water was the low-impedance fluid). Two initial fluid configurations were established: (1) an unstable configuration with the low-impedance fluid located in the central region of [−W/4, W/4] and the high-impedance fluid in the side regions of [−W/2, −W/4] and [W/4, W/2]; and (2) a stable configuration with the high-impedance fluid located in the central region and the low-impedance in the side regions [19].

COMSOL Multiphysics 6.1 (Burlington, MA, USA) was applied for the numerical simulation of SAW-induced inhomogeneous fluid motion at an exciting frequency of 6.65 MHz with a corresponding wavelength of 600 μm. The pressure acoustics–frequency domain, thermoviscous acoustics–frequency domain, laminar flow, transport of diluted species, and acoustic–thermoviscous interaction modules were fully coupled to capture the SAW-induced motion of inhomogeneous fluids. The numerical implementation was validated through systematic mesh refinement studies following established protocols in SAW microfluidics [23,24]. The mesh was successively refined from coarse (40,000 elements) to fine (320,000 elements) configurations. Convergence was quantified using the relative mesh convergence parameter *C*(*s*) for the mass distribution [19]:

(5) $$C(s)=\sqrt{\frac{\int_{\Omega }\;{\left(s-s_{ref}\right)}^{2}dS}{\int_{\Omega }\;{\left(s_{ref}\right)}^{2}dS}},$$

where Ω denotes the microchannel domain; *s* is mass concentration of the background fields; and *s_ref_* presents the solution obtained with the finest mesh (i.e., 320,000 elements for the microchannel width at full wavelength). Satisfactory convergence was achieved for *C*(*s*) < 0.01. The relative error exhibited exponential asymptotic behavior with mesh refinement, confirming numerical stability [19].

## 3. Results

### 3.1. Homogeneous Fluid Motion

**Figure 2:**
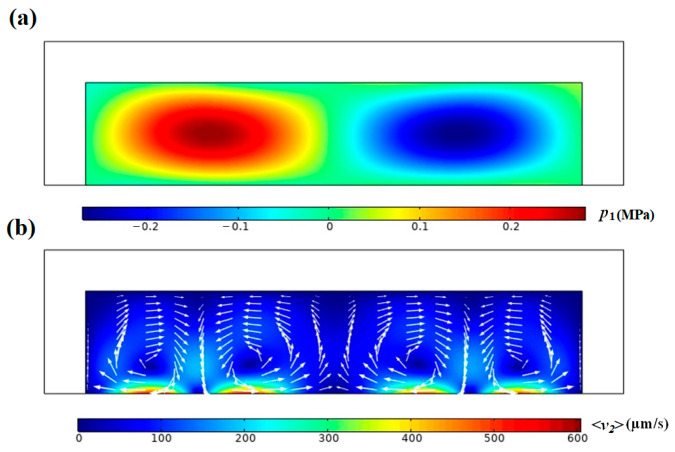
Simulated distribution of (**a**) first-order acoustic pressure (*p*_1_) and (**b**) time-averaged second-order velocity (<*v*_2_>) in arrows in the standing SAW field.

When the Ficoll PM70 solution is uniformly distributed in the microchannel, the first-order acoustic field *p*_1_ and the time-averaged second-order velocity field <*v*_2_> induced by the standing SAW are shown in Figure 2. A pressure node is established along the channel midline, with four Rayleigh stream rolls forming in parallel alignment. Notably, the streaming velocity exhibits significant vertical stratification, with substantially higher magnitudes near the floor compared to the ceiling. Adjacent rolls rotate in opposite directions at their interfaces, generating strong flow shear and steep velocity gradients. Our calculation agrees with previous numerical study under the same conditions [20].

### 3.2. Inhomogeneous Fluid Motion

**Figure 3:**
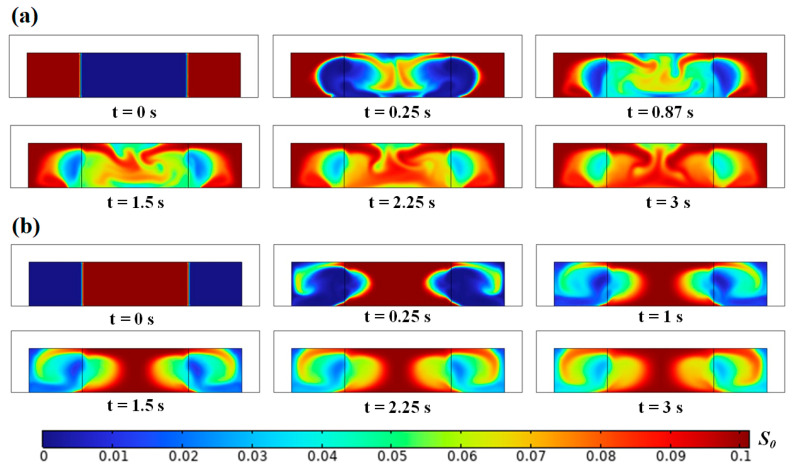
Inhomogeneous fluid flows in a microchannel driven by a standing SAW under the initially (**a**) unstable and (**b**) stable configurations.

The motion of inhomogeneous fluid driven by SAW with an initial Ficoll mass fraction of 0.1 (*s*_0_ *=* 0.1) is shown in Figure 3. Under the initially unstable configuration, a fraction of the high-impedance fluid migrates toward the acoustic pressure node through the microchannel ceiling, subsequently descending toward the floor, while the majority retracts toward the side walls. This bifurcation results in the formation of two elliptical regions dominated by low-impedance fluid. Rapid homogenization occurs near the microchannel floor due to strong convection effects, whereas the process is slow at *y* = ±*W*/4 where the local velocities are minimal. In contrast, only a small amount of high-impedance fluid migrates from the microchannel center toward the side walls through the ceiling under the initially stable configuration, with the majority remaining concentrated around the acoustic pressure node. Interestingly, at *z* = *H*/2 and *y* = ±*W*/4, the high-impedance fluid gradually contracts inward, again forming two symmetric ellipses of low-impedance fluid. Ultimately, the entire channel tends toward homogeneity due to the unstable flows at the microchannel ceiling and inward migration of high-impedance fluid. Overall, while SAW could partially counteract gravitational effects, it fails to achieve complete and stable fluid relocation. Nevertheless, its homogenization speed notably exceeds that observed in BAW-driven systems [19].

### 3.3. Inhomogeneous Fluid Relocation

**Figure 4:**
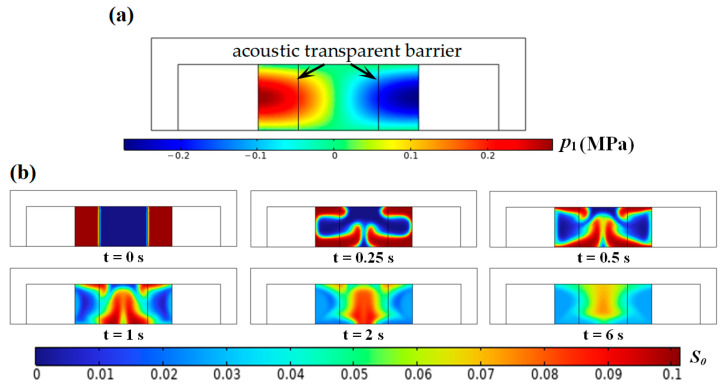
(**a**) Diagram of a partial standing SAW field in a microchannel and (**b**) motion of inhomogeneous fluid flow under the initially unstable configuration.

The foregoing results suggest that vertically nonuniform acoustic pressure distribution in the standing SAW field constitutes the primary impediment to successful relocation. To validate this hypothesis, two acoustically transparent barriers (e.g., thin membranes) are introduced at the acoustic pressure anti-nodes, effectively isolating the central half-wavelength region for simulation (see Figure 4). Under the initially unstable configuration, the high-impedance fluid forms vortexes near the floor and then migrates toward the acoustic pressure node at the microchannel midline, resembling the relocation pattern observed in BAW-driven systems. However, because of the vertically nonuniform acoustic pressure distribution and strong Rayleigh stream rolls near the floor, discrepancies persist between SAW- and BAW-driven flow patterns. Specifically, SAW produces a higher mass fraction at the ceiling, unlike the pyramid-shaped profile observed with BAW [19]. This corroborates our earlier conclusion that streaming velocity plays a dominant role in determining the efficiency of relocating inhomogeneous fluids.

### 3.4. Inhomogeneous Fluid Motion in a Half-Wavelength Microchannel

**Figure 5:**
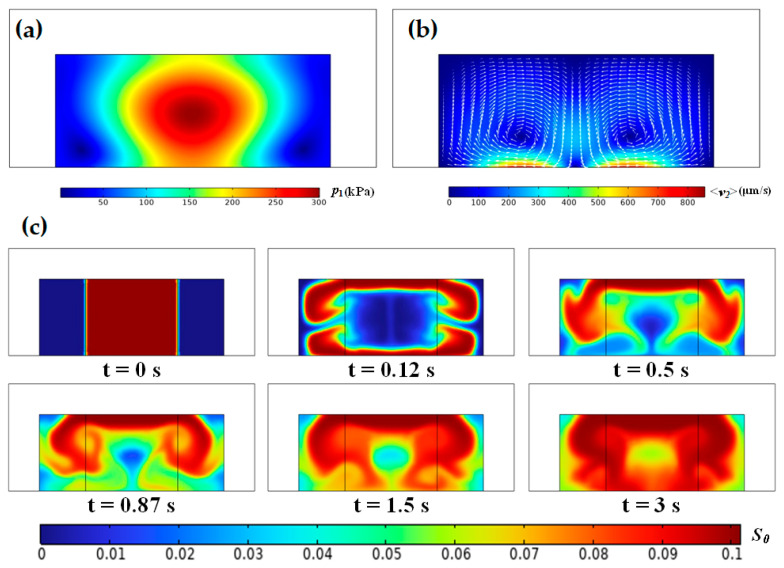
Simulated distributions of (**a**) acoustic pressure (*p*_1_), (**b**) time-averaged second-order velocity (<*v*_2_>), and (**c**) motion of inhomogeneous fluid in a half-wavelength microfluidic channel with the acoustic pressure anti-node in the midline under initially stable configuration.

To further elucidate the influence of the acoustic field on the inhomogeneous fluid flow, the channel width is reduced from 600 μm to 300 μm (half-wavelength). Correspondingly, the inhomogeneous fluid is bisected in width. In this configuration, a single pressure anti-node exists at the microchannel midline (see Figure 5). Under the initially stable configuration, high-impedance fluid migrates toward the corners, while low-impedance fluid remains centralized. Enhanced streaming near the microchannel floor accelerates homogenization in this region relative to the ceiling. Finally, an elliptical mass distribution emerges, characterized by low-impedance fluid at the center and high-impedance fluid around the periphery.

**Figure 6:**
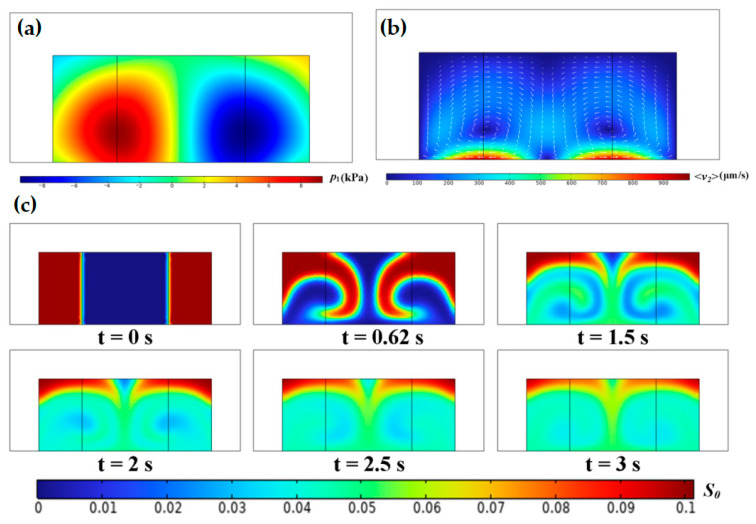
Simulated distributions of (**a**) acoustic pressure (*p*_1_), (**b**) time-averaged second-order velocity (<*v*_2_>), and (**c**) motion of inhomogeneous fluid in a half-wavelength microfluidic channel with the acoustic pressure node at the midline under the initially unstable configuration.

Under the initially unstable configuration with an acoustic pressure node at the microchannel midline, SAW-induced fluid mixing phenomena are observed (see Figure 6). High-impedance fluid moves from the low acoustic pressure zone at the ceiling toward the pressure node and then descends to the microchannel floor. Enhanced circulation and mixing arises from elevated flow speeds near the floor, although some high-impedance fluid still remains trapped at the ceiling.

## 4. Discussion

BAW-based acoustophoresis has been extensively investigated for microparticle accumulation/separation, yet its strong size dependence remains an intrinsic limitation of all volumetric-force techniques. When two fluids of different acoustic impedance flow side-by-side, the ARF acts normal to their interface and can induce relocation of inhomogeneous fluids. Systematic exploration of this phenomenon could improve the efficacy of acoustophoresis. Because the critical particle size in the acoustofluidics inverts with frequency [9], SAW operating at 10–1000 MHz exerts ARF that dominates over streaming drag for sub-micro particles, rendering SAW an ideal platform for high-resolution, high-throughput manipulation. In addition, the high propagating speed of SAWs along piezoelectric substrate and their rapid leakage into the adjacent liquid permit wider channels and therefore higher flow rates than those achievable with conventional BAW resonators. Here, we numerically explore whether a standing SAW field can replicate the relocation phenomenon previously demonstrated using BAW. Displacement boundary conditions were imposed on the floor of a PDMS microchannel with widths corresponding to a full and half a wavelength. High-impedance fluid (10% Ficoll PM70) migrates toward the pressure node, but the vertical distributions of acoustic pressure and intense acoustic streaming near the floor that are characteristics of a standing SAW field promote rapid homogenization before a steady relocated state is reached. Consequently, a classical “pyramid” or centered band pattern routinely observed in BAW devices cannot be sustained; instead, the system rapidly evolves into an elliptical low-impedance core surrounded by higher-impedance fluid. Nevertheless, the distinctive flow patterns generated by SAW induce excellent transverse folding and therefore offers an intrinsic mixing mechanism that is attractive for nanoparticle synthesis, pH-triggered polymer precipitation, or other reaction schemes requiring millisecond-scale blending [25,26]. Our findings provide valuable insights into the inhomogeneous fluid dynamics and advance the understandings of acoustofluidic mechanisms, with implications for the control of suspension concentrations in biomedical applications.

Acoustophoresis exploits ARF to concentrate, trap, wash, align, and separate cells [1,2,3]. However, its strong size dependency is a fundamental limitation of all volumetric force-based methods. ARF acting at the interface between two immiscible fluids arises from interactions between the incident and the scattered acoustic waves [27,28]. This force derives from the divergence in the time-averaged momentum flux density tensor, which is nonzero only at the interface. For BAW, acoustic streaming can be substantially mitigated by introducing an acoustic impedance gradient within the acoustofluidic microchannel, analogous to density gradient media employed in centrifugation and iso-electric focusing [8]. Specific differences in effective acoustic impedance drive spatial dispersion of the cell population toward their iso-acoustic point (IAP), where the acoustic contrast between the cell and surrounding liquid and the transverse displacement in the flow vanish. This size-insensitive approach enables continuous label-free analysis of individual cells. The relocation of high-impedance fluid from anti-node to node occurs when the acoustic force overcomes interfacial tension force, as predicted by stability theory and corroborated by microchannel experiments. An acoustic Bond number, defined as the ratio of relocation force (*F_rl_*) and interfacial tension force (*F_int_*), characterizes stable (*Bo_a_* < 1) and relocation (*Bo_a_* > 1) regimes [7]. Additionally, the critical acoustic energy density required for relocation can be significantly reduced by increasing the microchannel height, facilitating device optimization for handling immiscible fluids.

This study specifically addresses inhomogeneous fluids without suspended particles; that is, as stratified layers of miscible fluids with distinct acoustic impedances (Ficoll PM70 solution and deionized water). This differs from particle-laden suspensions where acoustic radiation forces act directly on discrete particles. The coupled behavior of particles within relocating fluids represents a subsequent complexity level [29], but this had been outside the scope of this fundamental investigation. Based on acoustic volume force density, inhomogeneous fluid exhibit behaviors analogous to particles in the acoustic field [30]. Therefore, high-impedance fluid migrates toward and accumulates at the acoustic pressure nodes as the particle’s motion under the Gorkov’s ARF [31]. However, prior research was focused on the BAW field. Our investigation revealed that, although high-impedance fluid moves toward the nodes with low acoustic pressure while low-impedance fluid flows toward the anti-nodes with high acoustic pressure in the standing SAW field, relocation of inhomogeneous fluid is impeded, mostly due to the vertically varying acoustic pressure and strong acoustic streaming flow near the microchannel floor. Unlike BAW-driven relocation, which initiates from the floor under gravity and acoustic pressure, SAW-driven flow preferentially originates from the ceiling and undergoes rapid homogenization at the microchannel floor. To validate this hypothesis, two cases were simulated: an anti-node and node located in the middle of a half and full wavelength-wide microchannel, respectively. In the first case, low-impedance fluid in the central region is pushed by the high-impedance fluid and subsequently distributes in an ellipsoidal shape, similar to the acoustic field distribution. In contrast, high-impedance fluid in the full wavelength microchannel forms vortices from the floor, moves towards the pressure node, and gradually becomes a stable configuration. Because of the strong acoustic streaming near the floor, concentration becomes higher at the ceiling, which is different from the pyramid shape in the BAW field [19]. Microfluidics is usually limited to diffusion-based mixing with a low efficiency because of low Reynolds numbers. Therefore, SAW may provide a superior alternative and efficient solution. Standing SAW was found more efficient in acousto-fluid force-driven fluid mixing compared to traveling SAW owning to double and single vortex flow through the channel, respectively [18]. A conductive liquid-based focused SAW (CL-FSAW) device could mix deionized water and fluorescent particle suspension rapidly and efficiently (e.g., 100 μL/min at an applied voltage of 21 V) for silver nanoparticle synthesis [32]. Integration of SAW and hyper-elastic channel walls could enhance the micromixing efficiency up to 0.99, modulate normal forces on the fluid, and reduce the pressure within the microchannel [33].

Our simulations reveal that SAW excitation parameters follow distinct scaling laws compared to BAW systems, with the vertical pressure field nonuniformity—characterized by displacement decay coefficient *C_d_*—fundamentally limiting relocation through exponential decay of the nodal plane from the substrate surface. This imposes three design constraints: channel height *H* should be minimized (*H*/*λ* < 0.1) for stratification or maintained at *H* ≈ *λ* for homogenization, though the latter increases viscous damping; streaming suppression via impedance matching or pulsed excitation is required to achieve controlled relocation rather than mixing, given that floor streaming velocities (~10 mm/s) exceed ARF-induced migration (~1 mm/s) by an order of magnitude; and half-wavelength configurations with low *H*/*λ* optimize mixing efficiency through elliptical concentration patterns and maximal interface stretching from ARF-streaming competition, whereas full-wavelength geometries produce four-roll structures favoring transverse transport.

This work has several limitations. Firstly, the simplified displacement conditions had been applied for the generation of SAW in order to reduce the computation burden. Therefore, the influence of the PDMS microchannel on the acoustic field is ignored. A more accurate simulation could be achieved using a full-size model incorporating solid mechanics and electrostatics modules, as previously proposed [34]. The wall thickness was found to affect the SAW propagation, energy coupling, and acoustic field inside the microchannel significantly. Furthermore, a fully coupled 3D model of boundary-driven acoustic streaming in the acoustofluidic devices based on the limiting velocity FEM is critical in understanding the underlying physical interactions from the electromechanical fields of the piezoelectric substrate to various acoustofluidic effects (acoustic radiation force and streaming-induced drag force) and fluid–solid interactions [24]. Such 3D models, despite being more time- and cost-intensive than 2D simulations, are essential for the design and optimization of novel microfluidics and complex on-chip configurations. Secondly, only the motion of inhomogeneous fluids has been considered here. For particle suspensions in inhomogeneous fluids, the coupled effect of acoustophoretic particle migration and fluid relocation leads to complex phenomena, including complete relocation without particle migration, acoustic migration without fluid relocation, simultaneous non-relocation and non-migration, and partial migration due to impedance contrast between particles, suspensions, and buffers [35]. The motion of dense suspensions is significantly different from the acoustic migration of a dense or dilute suspension or pure solutions in homogeneous fluids. Thirdly, this work is necessarily limited to numerical simulation due to the absence of requisite experimental infrastructure for 3D flow visualization. While our model incorporates validated physical parameters and mesh-converged solutions, experimental validation is essential to confirm these predictions. Specifically, three-dimensional, three-component (3D3C) measurements with astigmatism particle tracking velocimetry (APTV) [36] or confocal micro-PIV [37] would enable direct comparison of the predicted elliptical concentration patterns and streaming velocities. The characteristic flow signatures identified here—particularly the ceiling-initiated migration and floor-dominated homogenization—can provide experimentally testable hypotheses. Future work should also explore pulsed SAW excitation schemes that may decouple ARF-induced relocation from streaming-induced mixing, potentially enabling controlled stratification despite the inherent vertical nonuniformity of SAW fields. Finally, practical applications are required to evaluate the translation potentials.

## 5. Conclusions

Both BAW and SAW present mainstream tools for acoustofluidic manipulation, yet SAW offers superior spatial precision and high throughput. While BAW-driven relocation of inhomogeneous fluids has been theoretically and experimentally verified, the feasibility of achieving analogous phenomenon using standing SAW remains unexplored. Here, the first numerical study of SAW-induced motion of inhomogeneous fluids in microchannels with widths equal to either a full or half a wavelength has been performed. Our simulations reveal qualitatively distinct behaviors under initially stable and unstable configurations. Under the initially unstable configuration, only a fraction of high-impedance fluid reaches the acoustic pressure node before the entire system homogenizes, while most of them are swept to the side walls. In contrast, a small amount of high-impedance fluid migrates laterally along the microchannel ceiling under the initially stable configuration, while the central part contracts inward, again leading to rapid mixing. These phenomena arise from the vertically nonuniform pressure distribution and the strong acoustic streaming near the floor, characteristics inherent to the standing SAW field that we isolated through comparative analysis of half- and full-wavelength domains. Although a persistent, laterally relocated state cannot be sustained with a standing SAW, the resulting flow topology yields exceptionally fast transverse folding. This mechanism provides an attractive strategy for on-chip mixing, such as for nanoparticle synthesis and pH-jump precipitation. Ongoing experimental validation and systematic optimization of this approach are required for practical applications of such an acoustofluidic device.

## Figures and Tables

**Table 1:** Material properties and acoustic parameters at T = 25 °C in the numerical simulation [21,22].

Lithium Niobate (LiNbO_3_)		
speed of sound	*c_sub_*	4000 m/s
polydimethylsiloxane (PDMS)		
density	*ρ_w_*	920 kg/m^3^
speed of sound	*c_w_*	1076.5 m/s
Ficoll PM70		
density	*ρ_PM_* (*s*)	(1 + 0.349·*s*) × 996.85 kg/m^3^
speed of sound	*c_PM_* (*s*)	(1 + 0.167·*s*) × 1496.30 m/s
shear viscosity	*M_PM_* (*s*)	e^(10.82·*s*)^ × 0.893 mPa·s
diffusivity	*D_PM_* (*s*)	(1 − 5.51·*s* + 23.0·*s*^2^) × 1.21 × 10^−10^ m^2^/s
deionized water		
density	*ρ_DI_*	996.85 kg/m^3^
speed of sound	*c_DI_*	1496.30 m/s
shear viscosity	*μ_DI_*	0.89 mPa·s
bulk viscosity	*μ_b_*	2.47 mPa·s
compressibility	*κ*_0_	448 TPa^−1^
acoustic actuation parameters		
frequency	*f*	6.65 MHz
wavelength	*λ*	600 μm
displacement decay coefficient	*C_d_*	116 m^−1^
displacement amplitude	*d*_0_	0.1 nm

## Data Availability

The data presented in this study are available on reasonable request from the corresponding author.

## Abbreviations

The following abbreviations are used in this manuscript:

BAW	bulk acoustic wave
SAW	surface acoustic wave
FEM	finite element method
ARF	acoustic radiation force
GAF	gradient acoustic focusing
MEMS	mechanical–electrical micro-system
PDMS	polydimethylsiloxane
IAP	iso-acoustic point
CL-FSAW	conductive liquid-based focused SAW
3D3C	three-dimensional, three-component
APTV	astigmatism particle tracking velocimetry
